# Cognitive processing of a common stimulus synchronizes brains, hearts, and eyes

**DOI:** 10.1093/pnasnexus/pgac020

**Published:** 2022-03-10

**Authors:** Jens Madsen, Lucas C Parra

**Affiliations:** Department of Biomedical Engineering, City College of New York, 160 Convent Avenue, New York, NY 10031, USA; Department of Biomedical Engineering, City College of New York, 160 Convent Avenue, New York, NY 10031, USA

**Keywords:** intersubject correlation, brain–body connection, interpersonal physiological synchrony, engagement

## Abstract

Neural, physiological, and behavioral signals synchronize between human subjects in a variety of settings. Multiple hypotheses have been proposed to explain this interpersonal synchrony, but there is no clarity under which conditions it arises, for which signals, or whether there is a common underlying mechanism. We hypothesized that cognitive processing of a shared stimulus is the source of synchrony between subjects, measured here as intersubject correlation (ISC). To test this, we presented informative videos to participants in an attentive and distracted condition and subsequently measured information recall. ISC was observed for electro-encephalography, gaze position, pupil size, and heart rate, but not respiration and head movements. The strength of correlation was co-modulated in the different signals, changed with attentional state, and predicted subsequent recall of information presented in the videos. There was robust within-subject coupling between brain, heart, and eyes, but not respiration or head movements. The results suggest that ISC is the result of effective cognitive processing, and thus emerges only for those signals that exhibit a robust brain–body connection. While physiological and behavioral fluctuations may be driven by multiple features of the stimulus, correlation with other individuals is co-modulated by the level of attentional engagement with the stimulus.

Significance Statement Neural, physiological, and behavioral signals are synchronized between humans in a variety of settings. In this work, we show that physiological synchrony requires only 2 things, namely, effective cognitive processing of a common stimulus, and a robust coupling between brain activity and the physiological signal in question. We confirm this theory for heart rate, pupil size, gaze position, and saccade rate, as positive examples, and respiration and head movements as negative examples. We show that the strength of this correlation is co-modulated, i.e. correlation is modulated in unison for all signals in which correlation is detected. We propose that this common modulation is the result of attentional engagement with the stimulus.

## Introduction

It is well established that shared experiences can synchronize physiological signals between individuals. This has been observed for autonomic signals (1) including galvanic skin response, heart rate (HR), body temperature, respiration, as well as other signals such as gaze position (2) and pupil size (3). Synchronization of physiological signals between individuals has often been attributed to physical or social interaction (4–7). However, the simultaneous experience is not a prerequisite for physiological synchronization, as a number of studies have shown that physiological signals can be correlated between subjects even when they engage individually with dynamic natural stimuli (3, 8–11).

Stimulus-induced correlation between individuals has been studied extensively with neural measures including functional magnetic resonance imaging (fMRI) (12), electro-encephalography (EEG) (13), magneto-encephalography (MEG) (14), and functional near-infrared spectroscopy (fNIRS) (15) in particular during presentation of film (2, 12), auditory narratives (16, 17), and music (18). These studies show that subjects process narrative stimuli similarly, and that correlation of brain activity is predictive of memory of the narrative (17, 19). The cognitive state of viewers has been shown to influence the intersubject correlation (ISC) of neural activity. For instance, subjects that are attentive (20) and more engaged have higher ISC (21). In total, these studies indicate that perceptual and cognitive processing of the narrative are similar across subjects and depend on cognitive state.

In the context of physiological or behavioral signals, ISC is often referred to as “interpersonal synchronization,” implying that 2 or more people are co-present in a given context. A variety of mechanisms have been proposed to cause ISC such as social interactions (1), physical interactions (4, 7, 22), shared emotions (9, 10), and it has been argued that the strength of synchrony is modulated by empathy (23, 24), arousal (4, 25), attention (26), and more (1). The diversity of factors parallels the diversity of factors known to affect physiological signals. For instance, HR fluctuations are often discussed in the context of emotions, and pupil size in the context of arousal, although both are affected by a variety of other cognitive factors (27–33). We hypothesize, instead, that the cognitive processing of a shared stimulus is sufficient to induce ISC, thus providing a more parsimonious explanation for the variety of phenomena previously observed. We use the term “cognitive processing” in its general sense of creation and manipulation of mental representations, which includes stimulus perception (34). The hypothesis predicts that ISC is co-modulated in different signals, and importantly, that it will emerge only for physiological signals that exhibit robust coupling to the brain. If, instead, physiological synchrony is driven by a variety of factors, we would not expect a co-modulation of ISC nor would we expect signals with robust brain–body coupling to necessarily synchronize between subjects.

To test these opposing predictions we collected physiological, neural, and behavioral signals while participants watched informative videos. Data was collected individually for each participant to rule out effects of direct social interactions. Additionally, videos were selected to be engaging but not to evoke strong emotions or arousal as in previous studies on HR synchronization (6, 8, 9). These controls can falsify alternative hypotheses that require social or physical interactions (1, 4, 7, 22) or strong emotions or empathy (8–10, 23, 24). Our hypothesis also predicts that ISC should be modulated by attention and predictive of memory of the content in the video. To test for this, we used a secondary mental task that distracts attention from the stimulus (20) and subsequently probed for recall memory (35). We measured ISC for each individual (12, 13), and resolved it on the group level also across time and frequency.

As predicted, we found significant ISC between individuals only for those signal modalities that exhibit a robust brain–body connection, namely, gaze position, pupil size, HR, and saccade rate. We did not find significant correlation for respiration or head velocity, which indeed did not exhibit a robust coupling with brain activity. Consistent with our hypothesis, the strength of ISC co-varied across signal modalities, was modulated by attention and was predictive of recall. These results suggest that ISC is modulated in unison by the level of attentional engagement with the stimulus.

## Results

**Fig. 1.**
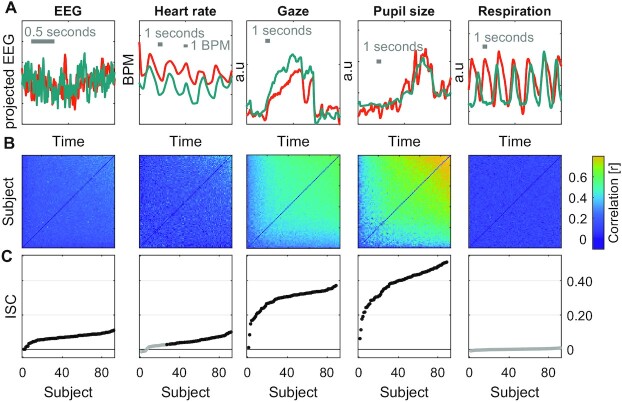
ISC of neural, physiological, and behavioral signals during passive video watching. (A) Signals for each of the modalities simultaneously recorded during Experiment 1. The 2 subjects shown (green and orange) have the highest ISC values measured for each modality. The EEG signal is the first component extracted from the 64-channel EEG using correlated component analysis. The gaze position signal is the horizontal gaze position. (B) Pearson correlation matrix between all pairs of the 92 subjects for each of the modalities. Subjects are sorted by increasing average correlation values. The correlation matrix for gaze position is the average correlation of gaze position in the horizontal and vertical direction. The correlation matrix for EEG is the sum of the correlation values obtained for 9 components extracted with correlated component analysis. (C) ISC values are the average of pairwise correlations for each subject, i.e. the mean over column of the correlation matrix in (B), excluding the diagonal, and averaged over the 3 videos presented (10 minutes total duration). Subjects are ordered by their ISC values (same as in B). Filled points indicate statistically significant ISC values and nonfilled points indicate they are not statistically significant. Statistical significance is determined using circular shuffle statistics (10,000 shuffles and corrected for multiple comparisons with FDR of 0.01). Circular shuffle means that the signal of each subject is randomly shifted in time, thus removing any intersubject relation.

To establish the strength of ISC in different modalities we presented instructional videos while simultaneously recording neural, behavioral, and physiological signals. In the first experiment with 92 subjects (Experiment 1) we recorded the EEG, HR, gaze position (gaze), pupil size (pupil), and respiration. We chose 3 instructional videos related to physics, biology, and computer science, each 3–5 minutes long, with a total duration of 10 minutes. Signals were recorded at different sampling frequency for each modality but aligned in time across all subjects and modalities (Fig. 1A). For each signal modality we computed Pearson's correlation of these time courses between all pairs of subjects (Fig. 1B). For gaze position the correlation is computed separately for horizontal and vertical position and then averaged. For the 64 channels of EEG, we first extracted several components of the raw evoked potentials that maximally correlate between subjects (36), computed pairwise correlation between subjects for each component, and then took the sum of the correlation values. In each signal modality, ISC is defined for each subject as the average Pearson's correlation between that subject and all others (Fig 1C).

The first observation was that ISC is statistically significant in most subjects and modalities (black points in Fig. 1C), but also quite variable across individuals. Significant correlation was detected in all modalities except respiration. Comparing ISC across modalities, the most robust correlation was found for pupil size (ISC_pupil_: 0.06–0.51, mean M = 0.39, and SD = 0.09) and gaze position (ISC_gaze_ is in the range of 0.01–0.37, M = 0.29, and SD = 0.06). They are significantly larger from chance values for all 92 subjects tested. Robust correlation is also found for EEG (ISC_EEG_: 0.00–0.11, M = 0.07, SD = 0.02, and 92/92 significant). We found that HR also correlates between subjects, but to a lesser extent with several subjects not exhibiting significant correlation (ISC_HR_: −0.01–0.10, M = 0.04, SD = 0.03, and 61/92 significant). For respiration we were not able to detect significant correlation between any participant and the group (ISC_resp_: −0.01–0.01, M = 0.00, and SD = 0.00).

### ISC co-varies across different modalities

**Fig. 2.**
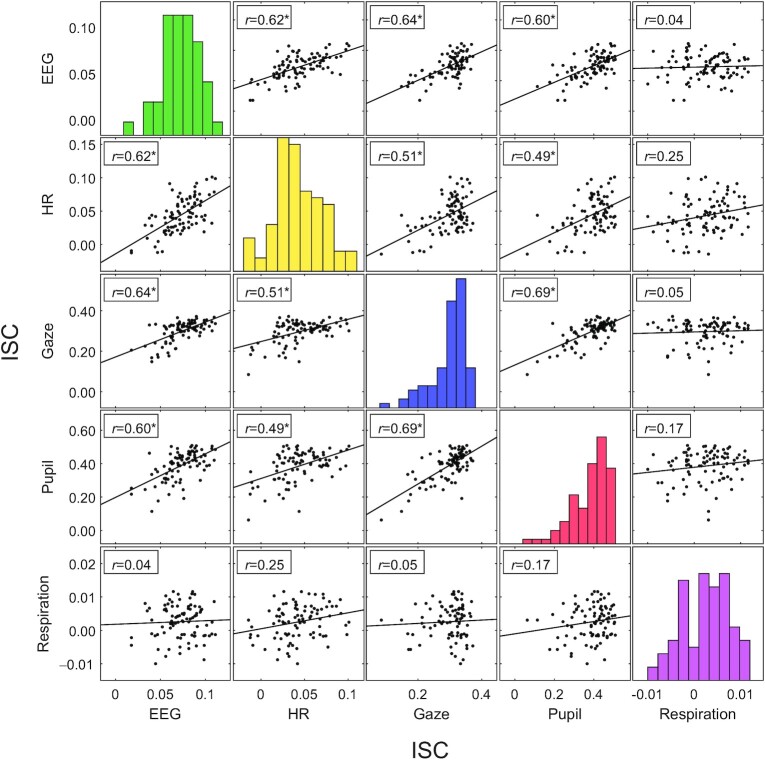
ISC is co-modulated in different signal modalities. ISC computed here for multiple signals recorded on 92 subjects while they watched instructional videos (same as in Fig. 1). Each point represents ISC between 1 individual and the group. The diagonal of the plot matrix is the histogram of each modality. Significant Pearson's correlation of ISC between each modality is indicated by a * (*P* < 0.01, bonferroni corrected). Lines indicate the linear least-squares prediction of vertical from horizontal axis (while points are the same in upper and lower triangles after flipping axes, prediction lines are not).

Given the strong variation of ISC across subjects, we wanted to determine if it co-varies in different signal modalities, i.e. if ISC is high in 1 modality, is it also high in other modalities? To this end we compared the ISC of different modalities across subjects (Fig. 2). Subjects with high ISC in EEG, HR, gaze position, and pupil size also showed high correlation in the other signal modalities. Correlation of ISC across subjects between these 4 modalities was significant in all cases (*r* = 0.49–0.69, *P* < 0.01 bonferroni corrected, and *N* = 92). However, we did not find any significant relation between the ISC of these 4 modalities and respiration (*r* = 0.04–0.25, *P* > 0.1 bonferroni corrected, and *N* = 92). The observation that the level of ISC of gaze position, pupil size and EEG co-varies across subjects is perhaps expected as they may all be driven by the visual dynamic of the video. What is more surprising is that high ISC in these modalities also coincided with high ISC of HR fluctuations, which thus far has been mostly attributed to emotional aspects of a stimulus 8 and would not be expected to be driven by visual dynamics of the video.

### Time scale of correlated signal fluctuations and co-modulation across time

**Fig. 3.**
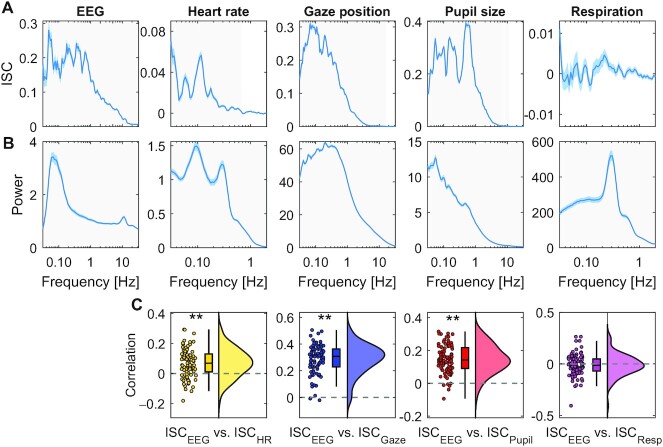
Time scales of signal fluctuations. (A) Frequency-resolved ISC: intersubject coherence spectrum computed by first band-pass filtering signals at different frequencies and then computing ISC averaged over videos and subjects. Band-pass filtering used center frequency on a logarithmic scale and a bandwidth of 0.2 octaves. Blue shading indicates SEM across *N* = 92 subjects. Significance of ISC values above 0 are established in each band using *t* test, corrected for multiple comparisons using 1D cluster statistics (light gray area indicates *P* < 0.01, cluster corrected). (B) Magnitude of fluctuations captured by the power spectrum, i.e. the power of the band-pass filtered signals. (C) Correlation between time-resolved ISC of EEG and time-resolved ISC of different modalities computed separately for each subject. Time-resolved ISC is computed in 10-second time windows with 50% overlap. Each dot is a subject. ** indicate *P* < 0.01 for a *t* test for nonzero mean ISC, uncorrected). The density is computed using kernel density estimation using a Gaussian kernel. The box plot shows the median and 25th and 75th percentile. Data aggregated over ∼15 minutes of video of Experiment 1.

ISC captures whether subjects move their eyes in unison, whether their HR increases or decreases together, whether their pupils dilate or contract or whether they inhale and exhale simultaneously. Given that ISC is co-modulated between modalities, we wanted to know if these correlated fluctuations were due to similar entrainment with the stimulus. To investigate this, we resolved ISC by frequency band, i.e. the signals are band-pass filtered prior to computing ISC resulting in a coherence spectrum. These coherence spectra differed significantly between modalities (Fig. 3A). Therefore, co-modulation is not likely to result from entrainment to a specific frequency band, and instead may have been driven by more complex properties of the stimulus. While coherent fluctuations differed between modalities, they generally were slower than 10 Hz and are strong in the frequency band around 0.1 Hz. ISC might, therefore, be reliably measured on a time scale of 10 seconds. We additionally analyzed the power spectra, which quantifies the magnitude of fluctuations in different frequency bands (Fig. 3B). They differed significantly from the coherence spectrum. Therefore, coherence was frequency-specific and not just a result of the underlying dynamic. Given this diversity, it is possible that each signal modality was modulated by something different in the stimulus across time. We, therefore, asked whether ISC, from 1-time interval to the next, changes together in different modalities. Indeed, time-resolved ISC of brain signals computed on 10-second intervals correlate significantly with time-resolved ISC of gaze position, pupil size, and HR (with correlations in the range of *r* = −0.04–0.39), but not with respiration (Fig. 3C). This correlation of ISC between modalities over time was weaker than what we found across subjects. This suggests that on a short time scale (less than 15 minutes), ISC of different modalities may be driven by a diversity of factors in these video stimuli. Nevertheless, both across subjects and across time, we found that ISC of different signal modalities are co-modulated.

### ISC is predictive of an individual's memory performance

**Fig. 4.**
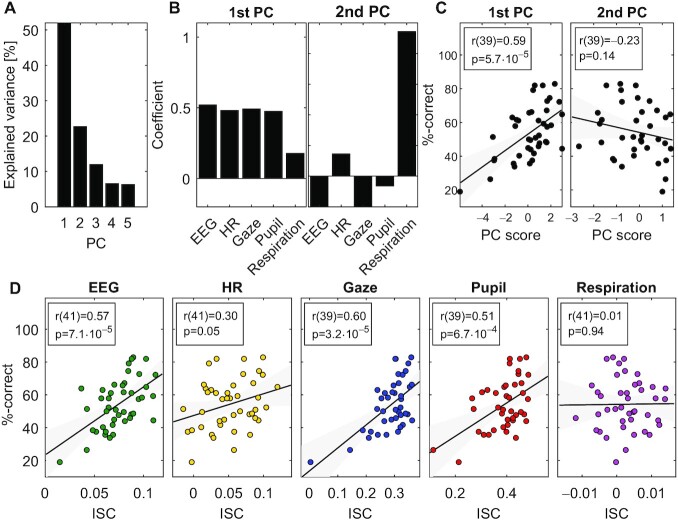
Common factor modulating ISC predicts memory recall: (A) variance explained by the principal components computed across all ISC modalities. (B) Loadings on the first 2 principal components, i.e. how much of each ISC modality is captured by each of the principal components. (C) The ISC projected onto the 2 first principal coefficients (PC Score) and the test taking performance of students (%-correct). (D) ISC of each modality and the test taking performance of students. Panels (A) and (B) include all data from Fig. 3 (Experiment 1) Memory performance for panels (C) and (D) was available only for a subset of 43 subjects. *P*-values are uncorrected.

We hypothesized that ISC is the result of cognitive processing, and thus we expected that the level of ISC will be predictive of memory performance. We, therefore, tested memory of the material in the video after presentation with a set of multiple choice questions (these data were available for 43 of the 92 subjects). Questions tested memory of the information presented in the video, which covered topics related to science, technology, and math (STEM). Questions probed for recognition and comprehension such as “What are stars made of?” or “How can Single Nuclear Polymorphism be useful?.” First, to quantify the common factor that is driving the co-modulation of ISC across subjects, we used principal component analysis (after z-scoring ISC values for each modality). The first principal component captured 52% of the population variance in ISC and the second component only 22% (Fig. 4A). The modalities captured by this first component were EEG, HR, gaze position, and pupil size (Fig. 4B), consistent with the co-variation of ISC observed in Fig. 2. Whereas the second principal component loaded mostly on respiration (Fig. 4B). As predicted, we found a strong correlation of memory performance with the first principal component of ISC (Fig. 4C) (r(39) = 0.59, *P* = 5.7⋅10^−5^, gaze position data was missing in 2 of the 43 subjects). In contrast, there was no significant correlation with the second component and test taking performance (r(39) = −0.23, and *P* = 0.14).

This result was confirmed when analyzing each modality independently (Fig. 4D and replicated for experiments 2 and 3 in Figure S5, Supplementary Material). ISC of EEG was predictive of students' test taking performance (*r*(41) = 0.57, and *P* = 7.1⋅10^−5^) as well as gaze position (*r*(39) = 0.60 and *P* = 3.2⋅10^−5^), and pupil size (*r*(39) = 0.51 and *P* = 6.7⋅10^−4^). To a lesser extent this was also true for HR (*r*(41) = 0.30 and *P* = 0.05). In contrast, ISC of respiration did not correlate with memory performance (*r*(41) = 0.01 and *P* = 0.94). We also analyzed the difference in ISC between men and women and did not find any significant difference in the modalities tested here (Figure S7, Supplementary Material).

### Correlation with others depends on the individual's attention to the stimulus

**Fig. 5.**
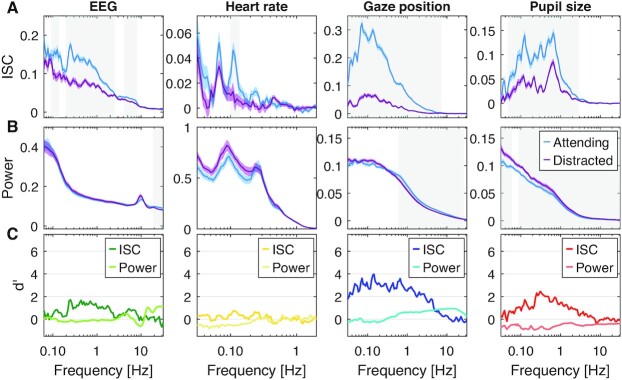
Attentional modulation of ISC and Power resolved by frequency. Here, subjects watch instructional videos (31 minutes total duration, Experiment 2) attending normally (blue) or distracted (purple) by performing a mental arithmetic task. (A) Coherence spectra measure ISC resolved by frequency. The color shaded areas indicate SEM across subjects. Significant differences between attending and distracted conditions are established for each band using a *t* test, corrected for multiple comparisons with 1D cluster statistics (gray-shaded frequency range indicates *P* < 0.01, *N* = 29, and cluster threshold corrected). (B) Power spectra measure power of signal fluctuations resolved by frequency. Shaded areas as in B. (C) Effect size is measured as Cohen's d’ with variance estimated across subjects. ISC and power are computed on band-pass filtered signals (as in Fig. 3.) and averaged over 6 videos.

The interpretation that cognitive processing of a common stimuli is required for ISC to emerge is consistent with the observation that it is modulated by attention for many of these modalities. Specifically, when subjects are distracted from the stimulus, ISC drops significantly for gaze position, pupil size, EEG as well as HR (3, 11, 20). To compare these effects across modalities and determine their time scales, we performed a new experiment in which *N* = 29 subjects watched videos while normally attending, and then again while distracted by a mental arithmetic task. In this Experiment 2, we used 6 instructional videos with a total duration of 31 minutes. We computed ISC again resolved by frequency but separately for the attentive and distracted conditions (Fig. 5A replicated for Experiment 3 in Figure S6, Supplementary Material). We found that ISC is significantly weaker in the distracted condition for all modalities (respiration was not measured in this experiment). We also analyzed the power spectrum of these signals to determine if the attentional effects are reflected in the dynamic of these signals (Fig. 5B). We found generally weaker effect sizes for the power spectrum (Fig. 5C). This suggests that attention does not strongly affect physiological dynamics, but rather, fluctuations align in time across subjects when attending to the stimulus and not otherwise. Finally, we analyzed traditional measures of arousal involving pupil size and HR (Figure S4, Supplementary Material). Only HR variability (HRV) was meaningfully modulated by attention.

### ISC occurs only for physiological signals that are coherent with brain signals

**Fig. 6.**
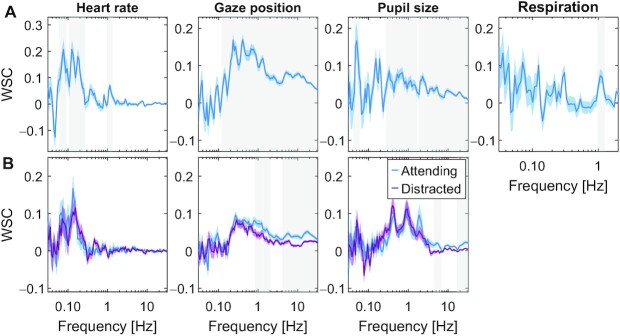
Within-subject coupling between EEG and other signal modalities during viewing of videos. WSC between EEG and gaze position, pupil size, HR, and respiration. The WSC is computed as the correlation between each signal modality and spatially filtered EEG. The spatial filters are found as the least square fit of scalp electrode potentials with the respective signal modality (computed separately for each frequency band; see supplement for variation across bands). (A) WSC computed by combining the 3 stimuli used in Experiment 1. Blue-shaded area indicates SEM over subjects. Gray-shaded frequency-band indicates significant difference from 0 (*P* < 0.01, *N* = 92 subjects, and cluster corrected *t* test on test data). (B) WSC values are computed separately for the attentive and distracted viewing conditions. WSC values are the average across the 5 stimuli used in Experiment 2 with *N* = 29 subjects, and the shaded area around the average WSC values is the SEM over subjects. Gray-shaded frequency range indicates a significant difference between attending and distracted conditions (*P* < 0.01, paired *t* test *N* = 29, and cluster corrected).

Given our hypothesis, we predicted that significant ISC in the physiological signals occurs if, and only if the signal correlates with brain activity within subjects during video watching. In this view, when brains correlate, so will other physiological signals that are driven by cognitive processing in the brain. The alternative hypothesis is that the brain–body connection itself is modulated by attention for these modalities. To test for this, we measured the within-subjects coupling between EEG and HR, pupil size, and respiration (Fig. 6). Specifically, at each frequency band we extracted a component of the EEG that best correlated with the respective signals. Here, we report the strength of this correlation, which we call within-subject correlation (WSC). We found that gaze position, pupil size, and HR significantly correlated with brain signals in some frequency bands (Fig. 6, and Figure S3 (Supplementary Material) for distribution over the scalp). This is trivially expected for gaze position due to saccade-evoked potentials (37), but this has not been previously reported for pupil or HR. Importantly, we found no correlation between brain potentials and respiration. We found only minor differences between the attend and distract conditions in these coherence spectra (for respiration we only have data on the attentive condition). The picture that emerges, therefore, is that the brain correlates with other subjects when it engages with the stimulus, and that this carries over to physiological signals as a result of an endogenous brain–body coupling, which is relatively stable with regards to attentional state during passive video watching.

### Brain–body connection predicts ISC for novel signals

**Fig. 7.**
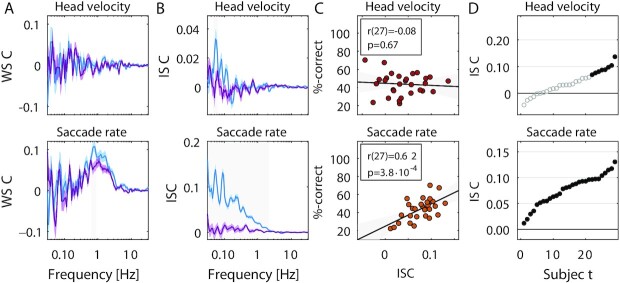
Predictions and test for saccade rate and head velocity. Here, using data from Experiment 2 from 29 subjects we test predictions on saccade rate and head velocity. (A) EEG has significant WSC with saccade rate but not head velocity, and neither difference with attentional state (attend: blue, distract: purple). (B) Significant ISC is observed for all subjects for saccade rate (29/29) but only a subset of subjects for head velocity (8/29). (C) Saccade rate, but not head velocity are predictive of memory performance across subjects. (D) Attention modulates ISC of saccade rate but not head velocity. Saccade rate is computed similarly to HR. Head velocity is calculated as the magnitude of the Hilbert transform of head position, combining horizontal, vertical, and depth directions with Euclidean norm. Significance established using the same methods as above.

Our theory, therefore, is that signals correlate between subjects, but only if there is a robust coupling between brain signals and the physiological or behavioral signal in question (Fig. 7A). We tested this on 2 additional signal modalities that have independently been proposed as markers of arousal. One is head velocity (38), following the basic notion of arousal as movement of the body. The other is saccade rate, which has been recently linked to effort and task engagement (39). First, we measured whether either are coupled to the EEG (on data from Experiment 2), and found this to be the case for saccade rate but not head velocity. We, therefore, predicted that saccade rate and not head velocity will be correlated across subjects and this correlation will be modulated by attention and predict memory. These predictions were all confirmed by subsequent analysis (Fig. 7B and 7C), and indeed, consistent with this theory, we found a co-modulation of ISC between all modalities across subjects (Figure S1, Supplementary Material) and across time (Figure S2, Supplementary Material). Note that correlated gaze position does not necessarily imply correlated saccade rate, as it only captures the number of saccade per unit time. On the other hand, head velocity could have been expected to correlate as large saccades tend to be accompanied by corresponding reorienting of the head (40). Therefore, these positive and negative examples were not trivially anticipated without the proposed theory.

## Discussion

**Fig. 8.**
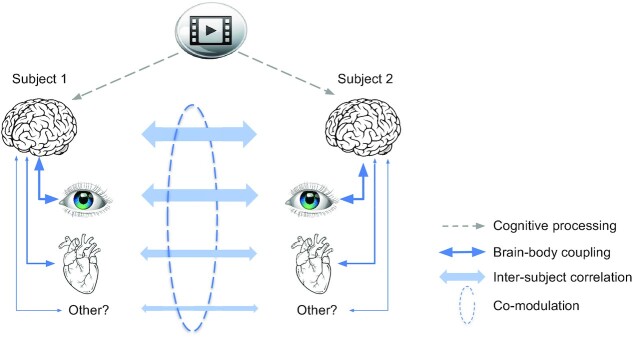
Schematic summary of results and proposed common mechanism. A video stimulus is processed cognitively by the brain, similarly in different subjects (dashed arrows—indicate cognitive processing encompassing perception and cognition; obviously signals enter the brain through eyes and ears). This cognitive processing causes similar fluctuations in signals of the body that exhibit robust brain–body coupling (solid arrows). Therefore, brain, physiological, and behavioral signals correlate between subjects (bold arrows). Subjects' attention variably engages with the stimulus, such that cognitive processing as a mediator of common fluctuations varies, across subjects and time. This variation results in a co-modulation of ISC in different signals. Links indicated in blue have been measured in this study as correlation. Narrow arrows are hypothesized causal effects (note that brain–body links may be bidirectional, which is obvious for the eyes and is discussed below for the heart as well).

To summarize, during viewing of informative videos we found significant ISC in all physiological signals that exhibited robust coupling with the EEG. This was the case for HR, pupil size, gaze position, and saccade rate, but not respiration or head velocity. This is consistent with the theory that correlation between individuals is the result of cognitive processing of a shared stimulus, but will only be observed for those physiological or behavioral signals that exhibit robust coupling with brain activity (Fig. 8). Importantly, the strength of ISC was co-modulated across signals, was predictive of an individual's memory performance and was attenuated when subjects were distracted from the stimulus.

The strength of ISC observed here as well as the modulation with attention and correlation with memory performances are in line with previous reports for HR (11), gaze position, pupil size (3), and EEG (17, 20, 35). The novel finding is that ISC is co-modulated in these signals across subjects and across time. We provide a novel theory as to which signals do or do not correlate and confirm this with saccade rate and head velocity as positive and negative examples.

The co-modulation observed here is surprising given the diversity of factors that have been previously proposed to underlie interpersonal physiological synchrony. Leaving out brain signals, this includes emotions (HR (5–7, 10)), arousal (HR (4, 25) and head velocity (38)), empathy (HR (6, 8, 24), skin conductance (23)), attention (eye movement (3) and HR (25, 26)), auditory features (HR and respiration (41, 42)), visual features (gaze position (43, 44)), physical movement (respiration (45, 46) and HR (22)), social interaction (HR (47)), and more. We argue more generally, that cognitive processing of the stimulus is a minimum requirement to evoke any of these constructs in a viewer's mind, and indeed, it suffices to explain the attention and memory effect we observed, namely, without processing the information imparted in the stimulus it is not possible to recall this information, and distracting the viewer will disrupt processing and thus reduce ISC. If interpersonal synchronization was really the result of the various factors as postulated in prior literature, it is not clear how this diversity of factors could have affected different modalities similarly, and even less that they should have modulated ISC in unison across time and across subjects. That said, the weak co-variation observed across time suggests that a diversity of factors may drive signal fluctuations differentially in each modality. Note that studies on interpersonal synchrony are often conducted with subject interacting, or at least co-present in the same space. These factors may have additional synchronizing effects (5, 7). What we showed here is that this is not a prerequisite for physiological synchrony.

An alternative interpretation of the present results is that low-level stimulus features are the source of co-variation in ISC. For example, visual dynamics may attract similar eye movement and this affects ISC of brain signals. With the strength of visual dynamics itself varying over time this could cause a co-modulation of ISC of the eyes and brain. There may be any number of stimulus features that guide visual exploration and could cause such co-variation in a bottom-up manner. However, this alternative interpretation does not explain the co-modulation of ISC observed across subjects. It also does not readily explain the co-modulation in heart and eyes, nor the observation that ISC of the heart is predictive of memory, a phenomenon that persists for audio-only narratives (11). In contrast, the effects of attention and on memory observed here are straightforward consequences of the cognitive processing hypothesis, which in our definition includes perceptual processing of the stimulus.

So, what might be the common factor that affects cognitive processing of the stimulus, and co-modulates the strength of ISC? We speculate that it is related to engagement with the stimulus. In previous work, with EEG during passive exposure to narrative audio and video we introduced the concept of “attentional engagement” with the stimulus (13, 17, 35, 48). We demonstrated that an objective behavioral measure of stimulus engagement correlates with ISC of the EEG measured on time scales of 10 seconds (49), presumably reflecting fluctuations of attention (20). In the vision literature, “attentional engagement” implies not only attracting attention (gaze) but also processing features of the target of attention (50–53). The notion that the ISC of EEG reflects a level of engagement with the stimulus has been adopted by a number of groups (21, 54, 55). The new finding here is that this engagement may be a common factor that also modulates the strength of fluctuations in HR, pupil size, saccade rate, and gaze position.

Regardless of the interpretation, this co-modulation of physiological responses is surprising for another reason—the dynamics of each of these signal fluctuations and their underlying neural control are quite diverse. For instance, neural control of HR and pupil size are ascribed to different brain structures. While pupil dilation has been most closely linked to activity in superior colliculus (SC) and locus coeruleus (LC) (29), HR is controlled by midbrain structures that are modulated by input from the amygdala, cingulate, and insula (27), although the LC also projects to nuclei involved in cardiac regulation (56). Additionally, the dynamics of these signals, as we have seen in their power spectra, differ considerably between modalities and the attentional effect on ISC manifests in different frequency bands for each. We note that the coupling between EEG and each of the signal modalities did not differ significantly with attentional state. Note also that the effects of attention on the power spectrum were generally small. Therefore, cognitive processing does most likely not substantially change the ongoing endogenous physiological dynamic within subjects. Instead, it appears to only time-align the existing dynamic to the external stimulus.

Our theory led us to prospectively analyse the correlation between EEG and various physiological signals. While a correlation of EEG with gaze position is well-established (in fact, it is considered a nuisance artefact (57)), to our knowledge this is the first study to report a correlation of EEG with HR, pupil size, and saccade rate. The theory also correctly predicted that saccade rate would correlate across subjects, something that has not been reported previously in the literature. Even less obvious was that this correlation of saccade rate would be modulated by attention and predict subsequent memory performance.

Here, we have focused on first-order correlation as we had no specific hypotheses related to nonlinear interactions. Such interactions can give rise to cross-frequency coupling in phase or amplitude and may warrant more complex analyses (58, 59).

We did not find ISC in breathing, which one might have expected. HR naturally increases when we inhale and decreases with exhale. This is known as respiratory sinus arrhythmia (60). Since HR synchronizes, we might have expected that breathing also does. Alternatively, in moments of suspense, viewers might be “holding their breath” in unison, which might have resulted in synchronized breathing. Breathing seems to align to the timing of a behavioral task (61), but here participants were not asked to do anything physical during viewing. Generally, studies that show correlated breathing involve some concurrent physical activity such as singing (46), speaking (62), or dancing (45) and correlation is not observed during passive listening to speech (63), though some effects have been found when listening to music (41). We do not report results on breathing rate, as it was not expected to fluctuate much during rest and would have required longer recordings. But we do not rule out that breathing rate may synchronize.

The fact that gaze positions are robustly correlated between subjects might not come as a surprise since eye movements are heavily driven by the dynamic of visual stimuli (64). We deliberately chose stimuli with compelling visual dynamics, such as animated graphics. A caveat of our analysis is that eye movements contaminate EEG signals, and thus ISC of eye movements could trivially result in ISC of EEG. However, we have previously shown that this confound can be largely ruled out when removing the EOG artefacts as we have done here (20). Furthermore, the co-modulation of ISC_EEG_ with other modalities is present even during audio-only narratives and in the absence of eye movements (in preparation). Another well-known artefact in the EEG is due to the heart beat itself. However, note that heart beat does not actually synchronize between subjects and so this artefact is not likely to contribute to the ISC of the EEG. Instead, the synchrony we detected between subjects is in the fluctuations of their HR. To our knowledge, the link we report here between EEG and HR has not been reported previously in the literature. One additional caveat is that at low frequencies (below 0.1Hz) the EEG may also include galvanic skin response from the scalp, however we still find ISC of EEG and WSC for signals above this frequency.

The pupil size is known to be affected by luminance, but also higher level factors such as problem solving and cognitive effort (31, 65), affective processing (33, 66), and attention (67). So, to see the pupil size correlated across subjects might be expected. That this pupil correlation should be modulated by attentional engagement is consistent with the argument that task engagement is reflected in pupil dynamics on similarly short time scales (39). Our most recent work suggests that this phenomenon is not contingent on luminance fluctuations as it appears also during audio-only narratives (in preparation). That the ISC of pupil should be co-modulated with ISC of HR was not expected from the literature. We have not analyzed eye blinks, however, given reports of a correlation of blinking across subjects during movies (68), we expect that this will behave similarly to the other measures we have taken from the eyes. Given the coupling that has been observed between EEG and gastric rhythms (69) we also predict that these will show significant ISC.

Pupil and HR have often been linked to arousal (see below), although the term “arousal” has quite diverging definitions in the literature. We expected that head velocity could serve as a marker of physical arousal (38), and thus may also correlate. Correlation may also be expected given the ISC of gaze position and the fact that large eye movements tend to be accompanied by corresponding head movements (40). We did not find significant ISC in head velocity in our smaller datasets. Consistent with that, ISC of head velocity did not significantly correlate with memory or attention, nor did head velocity significantly correlate with brain potentials. We did, however, find a weak ISC in the larger dataset of 92 subjects (see Supplement), and therefore, one might be able to resolve these effects on larger datasets. Indeed, our theory does not stipulate an all-or-nothing effect. A weak brain–body link may lead to similarly weak physiological ISC and related effects.

Correlation of HR between subjects has previously been linked to emotional processing of video stimuli (8, 9), which may not have been a main driving factor in our mostly informative videos. In social contexts, HR synchronization has also been linked to empathy (70) and social bond (71). We propose that cognitive processing of the natural stimulus is a requirement to infer the emotional valence of the film (9), for an audience to bond with a performer (4) or mother and child to interpret social cues (47). Thus, cognitive processing of the external natural stimulus is the common denominator, even for interpersonal HR synchrony. A caveat to this conclusion is that we did not look for other factors that could have driven the different modalities differently. All videos were designed to communicate factual information related to science and technology, and not, for instance, evoke suspense or strong emotions (5, 6, 10, 72). So it is possible that we did not find other factors simply because we did not manipulate other factors with our experimental paradigm. Future work should consider varying stimulus properties along different dimensions.

The cognitive control of HR is a crucial prerequisite for the co-modulation observed here. Indeed, central control is well-established and explains the effects of cognition on HR and HR variability (27, 28). For instance, HRV correlates with neural activity measured with fMRI (73). There is also evidence for the opposite causal direction, whereby volitional control of breathing affects HR and in turn neural activity (74). The heart beat itself seems to affect saccade timing (75), evoke EEG (76), as well as visual detection performance (77). Also, strong stressors can increase HR and at the same time enhance the magnitude of evoked responses in EEG (78). But none of these studies report a link between EEG and HR as we have found here, nor do they anticipate that ISC is co-modulated in these modalities

A recent theory proposes a common drive to pupils and saccades (39). The theory posits that this common drive is affected by a variety of cognitive processes, in particular “task engagement“, but authors distinguish this from ”arousal". This common drive is postulated to be the activity in SC. Indeed, the SC along with the LC are midbrain nuclei that mediate cognitive effects on the pupil (29, 79). A common drive of pupil and saccades reconciles the dependence of pupil dilations on arousal and effort as well as its fluctuations during a task. Consistent with this we find that pupil size and saccade rate both behave similarly with attention and memory and importantly the ISC in these 2 modalities are co-modulated. Note that the LC also connects to midbrain structures that control cardiac function (80) and may mediate cognitive effects on HR (56). Therefore, the LC is well-situated to mediate cognitive effects on pupil size, saccades rate, and HR.

In conclusion, this work brings together 2 separate research fields which have demonstrated correlation between subjects in ecologically valid scenarios, namely, interpersonal physiological synchrony (1) and ISC of neural activity (12). We postulate that cognitive processing of a natural dynamic stimulus is required to drive and coordinate behavioral responses of the body. Our rationale is that humans have evolved to quickly respond to the natural stimuli in their environment, and to do so, the brain processes information and prepares the body to react. This leads to rapid, but reliable fluctuations of HR, pupil size, gaze position, and others on the scale of seconds. If the brain is not processing the stimulus effectively, i.e. similarly across subjects, then the body will not respond appropriately and these fluctuations will no longer be guided by the stimulus. As the brain changes in attentional state on time scales of 10 seconds or longer the correlation induced across subjects is modulated for different modalities in unison. Although our study here is only correlational, we do postulate a causal effect of cognitive processing on physiological and behavioral responses that is similar across subjects and modalities. Evidently, there could be a bidirectional interaction. This is most obvious for eye movements, as our view can affect our cognitive state. A bottom-up effect has also been hypothesized for heart signals (74) and is the basis for some meditation practices that focus on breathing (81). Establishing a causal direction of these effects will be difficult but is a worthwhile topic for future research.

## Materials and methods

### Participants

A total of 158 subjects participated in 1 of 3 experiments, where they watched informative videos. In Experiment 1, *N* = 96 participated (51 Female, age 18–49, M = 25.33, and SD = 7.29; 4 subjects were removed due to bad timing or bad signal quality). In Experiment 2, *N* = 32 subjects participated (21 females, age 18–57, M = 25.93, and SD 8.94 years; 3 subjects were removed due to bad signal quality). Lastly in Experiment 3, *N* = 31 participated (19 females, age 18–50, M = 25.79, and SD = 8.13 years; 2 subjects were removed due to bad signal quality).

### Stimuli

A complete list of the video stimuli is provided in Table S1 (Supplementary Material). These stimuli have been used in previous studies (3, 11, 35). For Experiment 1, we selected 3 videos from YouTube channels that post short informal instructional videos, namely “Kurzgesagt—In a Nutshell” and “Minute Physics.” The videos covered topics related to physics and biology with a short duration (Range: 3–6.5 minutes and total duration 15.5 minutes). In Experiment 2, we selected 6 videos from “Khan Academy,” “eHow,” “Its ok to be smart,” and “SciShow,” which are popular online educational channels on YouTube. The videos covered topics related to biology and physics, with a short duration (Range: 4.5–6.5 minutes and total duration 31 minutes). In Experiment 3, we selected 5 informal instructional videos again from YouTube, covering topics related to physics, biology, and computer science with a short duration (Range: 2.4–6.5 minutes and Average: 4.1 +/− 2.0 minutes). Data on gaze position and pupil size for Experiment 2 have been previously analyzed (3), as well as data on HR from Experiment 3 (11). All other data and analyses are new to this study, i.e. all of the data from Experiment 1, EEG, HR, saccade rate, and head velocity from Experiment 2, and EEG, pupil size, gaze position, saccade rate, and head velocity from Experiment 3.

### Procedure

All experiments were carried out at the City College of New York with approval from the Institutional Review Boards of the City University of New York. Documented informed consent was obtained from all subjects at the start of the experiment. Subject watched the videos on a 19” monitor while seated comfortably in a sound-attenuated booth with white fabric walls and normal ambient LED lighting placed around the subject. Sound was played through stereo speakers placed at 60° angles from the subject next to the monitor, both at a distance of approximately 60 cm from the subject.

In Experiment 1, subjects watched 3 instructional videos while their EEG, electro-oculogram (EOG), electro-cardiogram (ECG), respiration, pupillary responses, gaze, and head position were recorded. In the second experiment, subjects watched 6 instructional videos while their EEG, ECG, pupillary responses, gaze, and head position were recorded.

In all the experiments, subjects were instructed to watch the videos normally as they would at home, while being relaxed and sitting still. We refer to this as the attentive conditions (A). After they had watched the videos, subjects were given a 4-alternative forced-choice questionnaire covering factual information imparted during the video (11–12 recognition questions per video; see Table S1, Supplementary Material). The videos and question pairs were presented in random order. In Experiment 1, subjects were not aware that they would be tested on the material, whereas in Experiments 2 and 3 the test was anticipated. After answering questions, in Experiments 2 and 3 subjects were asked to watch the videos again, but this time to silently count in their mind backwards in steps of 7 (starting from a prime number picked at random between 800 and 1,000). The second viewing with concurrent counting is referred to as the distracted condition (D).

For segmentation of the physiological signals we used common onset and offset triggers, in addition, a flash and beep sound was embedded right before and after each video, which were recorded using a StimTracker (Cedrus) to ensure precise alignment across all subjects. To enable all modalities to be on the same time scale, we used the lab streaming layer (LSL) protocol. In addition, triggers were sent to both the eye tracking and EXG recording systems, timestamps from each system were used in a linear regression model to convert timestamp between each modality estimated using the common triggers.

### Recording and preprocessing of EEG

The EEG was recorded at a sampling frequency of 2,048 Hz using a BioSemi Active Two system. Participants were fitted with a standard, 64-electrode cap following the international 10/10 system with the ground electrode located next to POz. In addition, the EOG was recorded with 6 auxiliary electrodes (1 located dorsally, ventrally, and laterally to each eye). The EEG and EOG data is band-pass filtered between 0.016 and 250 Hz by the Active two system prior to sampling. The signal was then digitally high-pass filtered (0.05Hz cut-off) and notch filtered at 60 Hz to remove line noise. To remove artefacts and outliers Robust PCA was used (82), and subsequently the signal was low-pass filtered (64 Hz cut-off) and down-sampled to 128 Hz. Bad electrode channels were identified manually and replaced with interpolated channels. The interpolation was performed using the 3D Cartesian coordinates from the electrode cap projected onto a plane using all surrounding “good” electrodes. The EOG channels were used to remove eye-movement artefacts by linearly regressing them from the EEG channels, i.e. least-squares noise cancellation (code for robust PCA and EOG noise cancelling in EEG can be found in https://www.parralab.org/isc/). In each EEG channel, additional outlier samples were identified as values exceeding 4 times the distance between the 25th and the 75th quartile of the median-centred signal, and samples 40 ms before and after such outliers were replaced with interpolated samples using neighboring electrodes.

### Recording and preprocessing of ECG

The ECG signal was recorded using 2 ECG electrodes placed below the left collar bone and 1 on the left lumbar region with a BioSemi Active Two system at a sampling frequency of 2,048 Hz. The ECG signal was detrended using a high-pass filter (0.5 Hz cut-off) and subsequently notch filtered at 60 Hz to remove line noise. Peaks in the ECG corresponding to the R-waves were found using *findpeaks* (built-in Matlab function). The instantaneous HR is computed for each beat as the inverse of time intervals between subsequent R-wave peaks as in (11). To ensure the same sampling frequency for all subjects this instantaneous HR signal is resampled at a regular sampling rate of 128 Hz.

### Recording and preprocessing of gaze position, head velocity, and pupil size

Gaze position, head movements and pupil size were recorded using the Eyelink 1000 eye tracker (SR Research Ltd. Ottawa, Canada) with a 35 mm lens at a sampling frequency of 500 Hz. Subjects were instructed to sit still while the experiment was carried out, but were free to move their heads, to ensure comfort (no chin rest). A standard 9-point calibration scheme was used utilizing manual verification. Stable pupillary responses were ensured by adjusting the background color of the calibration screen and all instructions presented to the subjects to be the average luminance of all the videos presented during the experiment. After each stimulus presentation, a drift-check was performed and the eye tracker was recalibrated if the visual angular error was greater than 2°. Blinks were detected using the algorithm of the eye tracker. These artefacts, blinks and 100 ms before and after were filled with linearly interpolated values. Head velocity is computed as the absolute value of the analytic signal of the Hilbert transform (root mean square summed over the 3 directions). Saccades were detected by the algorithm of the eye tracker. Instantaneous saccade rate was calculated as the inverse time interval between saccades and up-sampled to a constant sampling rate of 2,000 Hz to match the other eye tracking signals.

### Recording and preprocessing of respiration

The respiration signal was recorded at a sampling frequency of 2,048 Hz on the BioSemi Active Two system using a SleepSense 1387 Respiratory Effort Sensors, which captures the tension on a belt worn around the chest of the subject. The polarity of the signal was detected using peaks in the respiration signal and inverted to ensure the correct phase of the signal.

### ISC analysis of gaze position, pupil size, respiration, HR, saccade rate, and head velocity

For each of the gaze position, pupil size, respiration, HR, saccade rate and head velocity signals, the ISC was computed in the following 3 steps: (1) computing the Pearson's correlation coefficient between a single subject's signal (each of the 6 modalities independently) and that of all other subjects while they watched a video. Correlation is computed by summing over all time points of the video (with durations specified in Table S1, Supplementary Material) and then averages over all videos. (2) A single ISC value for a subject was obtained by averaging the correlation values between that subject and all other subjects. (3) The 2 first steps are then repeated for all subjects, resulting in a single ISC value for each subject. For ISC of gaze position, we compute the ISC in the horizontal and vertical gaze direction using the procedure as described above separately. To obtain 1 single ISC_gaze_ value, we average the ISC for the horizontal and vertical directions.

### ISC of EEG

For the EEG signals ISC was computed using correlated component analysis (36), with code available at http://parralab.org/corrca/. This method finds linear components of the EEG that are most correlated between subjects. The components consist of several projection vectors that linearly combine electrodes, on which the data is projected. The ISC of each component is obtained by computing the correlation coefficients of the projected EEG between each participant and all other participants. We only use components that are significantly correlated above chance (circular shuffle on test set data, see below). This yielded 3–9 components depending which of the 3 Experiments was analyzed. ISC values are then summed over all significant components.

### Statistical significance of ISC values per subject

To determine whether ISC values are significantly larger than 0 (Figs 1C and 7D), we determine the null distribution of ISC values on surrogate data obtained with circular shuffle statistics (83). *P*-value (type I error rate) is then the fraction of shuffles with ISC values larger than in the original unshuffled data. We performed 10,000 circular shuffles estimating *P*-values down to 0.0001. For EEG, the ISC values are measured on components that have been optimized to provide high ISC values. To avoid an upwards bias in ISC during statistical testing, components are optimized on training data and significance is established for separate test data (2 video clips are used for training and 1 for testing). Note that all statistical comparisons of correlation values, here and in the remainder, are performed by computing correlation values with the identical number of samples for the conditions to be compared.

### Frequency analysis of ISC (coherence spectrum)

We performed a frequency analysis to investigate at which time scale the recorded signals correlated between subjects. Each signal from each subject was band-pass filtered using 5th order Butterworth filters with logarithmic spaced center frequencies with a bandwidth of 0.2 of the center frequency. The ISC was computed for each subject in each frequency band for all videos. To obtain a single ISC value per frequency band we average ISC values for all videos and subjects.

### Computation of d-prime (d’) effect of attention on coherence and power spectrum

To determine the effect size of the attentional modulation for both the frequency-resolved ISC and Power spectrum we compute the d-prime statistics. For each frequency band (see section above) we compute the ISC and power for each subject in the attending and distracted conditions. We quantify the effect size between the 2 conditions as the difference of the means divided by the standard deviation across subjects of the differences (paired Cohen's d).

### Cluster statistics for difference between attentive and distracted conditions

To determine significant difference in spectra between attending and distracted conditions (for Figs 5A, 5B, 6B, 7A, and 7B), we use cluster shuffle statistics as follows. Since different frequency bands in the frequency-resolved ISC (and WSC) are not independent, we use 1D cluster statistics including random shuffles to correct for multiple comparisons following an established procedure (84, 85). Briefly, this procedure involves 4 steps: (1) take the difference between the spectrum in the attending and the distracted condition for each subject. (2) compute a one-sample *t* test on this difference for each frequency band. (3) clusters are identified as consecutive frequency bands with *P*-values below 0.01. The *t*-values within each cluster are then summed. (4) Run 10,000 permutations in which we randomly change on half of the subject's the sign of difference between the spectra computed in step 1. Steps 2 and 3 are then repeated while keeping the sum of *t*-values of the largest cluster. Finally, we compare the clusters’ *t*-values obtained in step 3 with the distribution of permuted cluster *t*-values obtained in step 4. Clusters with larger than 99% (corresponding to *P*-value < 0.01) of the permuted distribution were considered significant after multiple comparison cluster correction. Note that for the contrast in attention all data was used for optimization (of components of the EEG) including attentive and distracted conditions, so that any difference cannot be due to the optimization procedure.

### Cluster statistics for significant of intersubject coherence spectra

To determine if frequency-resolved ISC (and WSC) values are significantly different from 0 (Figs 3A, 3B, and 6A), we use a similar cluster statistic as above. For cases involving EEG (correlated component analysis and regression), we use test data to avoid upwards bias due to optimization, which is performed on separate training data. The shuffling and cluster correction procedure consists of the same 4 steps as above, except that we divide subjects in 2 equal size groups at random. The premise of this is that values around 0 will not differ significantly if placed at random in 2 different groups (84).

### WSC analysis (brain–body coupling)

To determine the coupling between the brain and different signal modalities we compute WSC using ordinary least squares regression, i.e. we measure how well each signal modality can be predicted linearly from the multidimensional EEG signal. Specifically, denote with ${x_i}( t )$ the EEG signal at time *t* for in channel *i*, and with $y( t )$ the signal of the modality of interest. The linear prediction is then $\hat{y}(t) = {\sum _i}{w_i}{x_i}(t)$ and we find weights ${w_i}$ such that ${\sum _t}{( {y( t ) - \hat{y}( t )} )^2}$ is minimized. Linear projections of this sort are conventionally referred to as components of the EEG (as in principal component analysis, or correlated component analysis used for the ISC). WSC is then the Pearson's correlation of $y( t )$ and $\hat{y}( t )$ extending over all stimuli. In the case where we differentiate between the attend and distract conditions, we compute component weights ${w_i}$ and WSC in each condition separately. For the frequency-resolved WSC, we band-pass filter the signal modality and EEG in the same fashion as the time-resolved ISC, namely using 5th order Butterworth filters with logarithmic spaced center frequencies with a bandwidth of 0.2 of the center frequency. To compute the WSC in the frequency resolved condition, we find components for each band separately. WSC in all instances was computed on test data using leave-one-subject out cross-validation (i.e. regression parameters are estimated including all subjects except for the test subject; WSC is then computed on that test subject; this train-test process is then repeated for all subjects.) Statistical significance is established in the same way as for ISC as described in the 2 previous sections.

### Common factor analysis of ISC

To find the common factor of ISC between signal modalities, we first remove outliers of the ISC data that are larger or smaller than 4 times the interquartile difference of the data. This was done since the standardization of the data is sensitive to outliers. We then standardize the ISC (0 mean and unit variance) and compute the principal components.

## Supplementary Material

pgac020_Supplemental_File
