# Supplementary material for: Cognitive processing of a common stimulus synchronizes brains, hearts, and eyes
Source: PNAS Nexus. 2022 Mar 10;1(1):pgac020. doi: 10.1093/pnasnexus/pgac020 (PMC9802497; doi:10.1093/pnasnexus/pgac020)
Supplement: pgac020_Supplemental_File [file pgac020_supplemental_file.pdf]

PNAS nexus

Supplementary Information for
Cognitive processing of a common stimulus synchronizes brains, hearts, and eyes

Jens Madsen, Lucas C. Parra

Corresponding author: Jens Madsen

Email: jmadsen@ccny.cuny.edu

This PDF file includes:

Supplementary text
Figures S1 to S7
Tables S1

Supplementary Information Text

Stimuli

Experiment	Title	Abbreviation	Duration	URL*	Topic
1,3	Why are Stars Star-Shaped?	Stars	3:28	VVAKFJ8VVp4	Physics
1,3	The Immune System explained	Immune	6:48	zQGOcOUBi6s	Biology
1,2	Are we all related?	Related	6.03	mnYSMhR3jCI	Biology
2	What if we killed all the Mosquitoes?	Mosquitoes	4.21	e0NT9i4Qnak	Biology
2	Dielectrics in Capacitors	Capacitors	5.46	rkntp3_cZl4	Physics
2	Work and the work-energy principle	Work energy	6.26	30o4omX5qfo	Physics
2	How do people measure Planets and Suns?	Planets	4.23	bYgV9nvgJ3E	Astronomy
2	What function does an Enzyme have?	Enzymes	4.29	IkRZKqDdwzU & AAZ03aScPr8	Biology
3	Why Do We Have More Boys Than Girls?	Birth rate	2:48	3IaYhG11ckA	Biology

3	How modern Light Bulbs work	Bulbs	2:57	oCEKMEeZXug	Physics
3	Who invented the Internet? and why?	Internet	6:32	21eFwbb48sE	Computer Science

Table S1. Experiment, title, abbreviation, duration, web address. *URL of videos beginning with https://www.youtube.com/watch?v=

Full list of questions and answer options can be found here.

Co-modulation of ISC across subjects and modalities

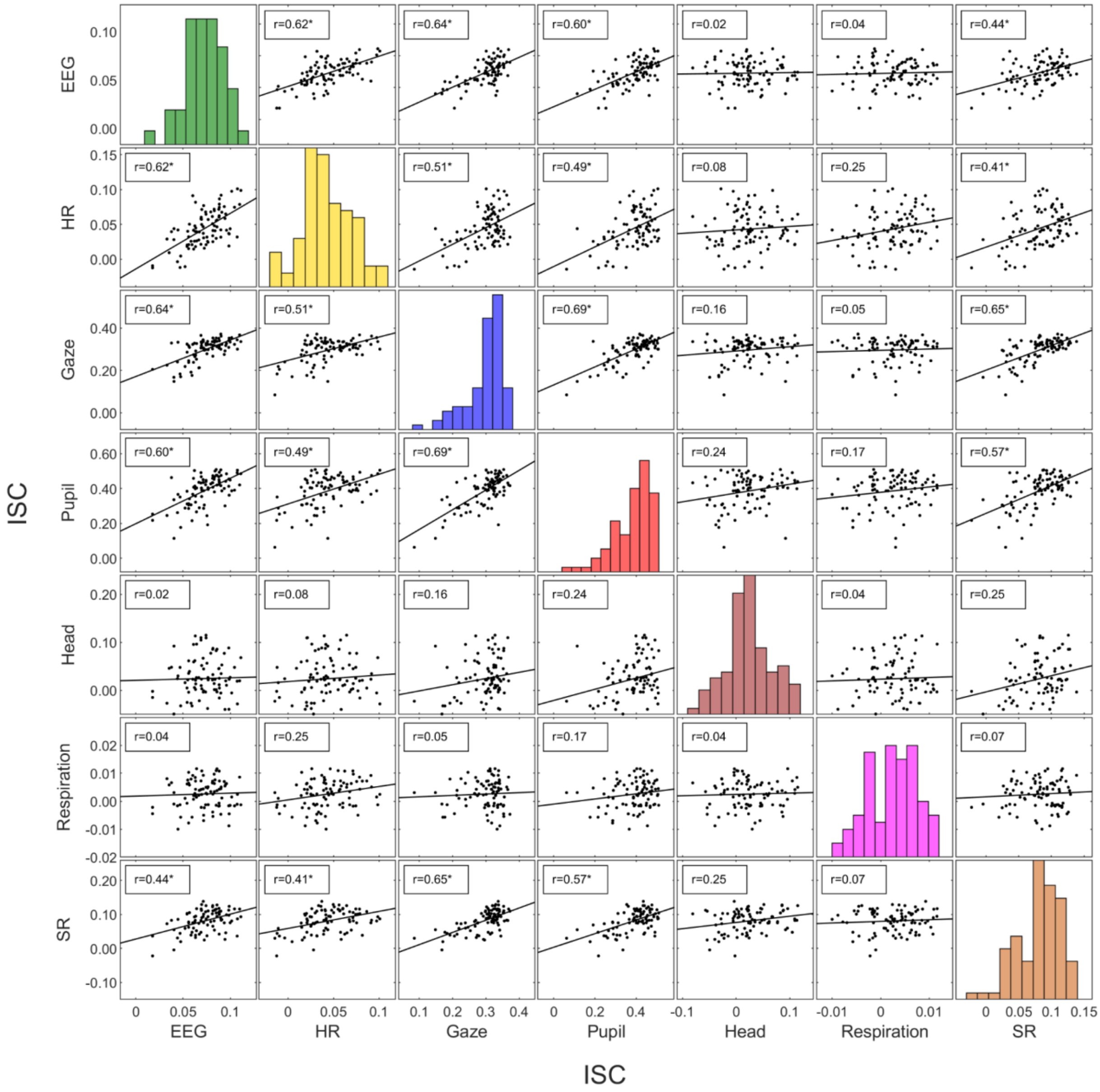

Figure S1: ISC of different modalities is co-modulated across subjects. ISC is computed here for each subject as in Fig 1 (Experiment 1). Here we are including head velocity (Head) and saccade rate (SR), modalities which we tested only after making a prediction based on Fig. 7.

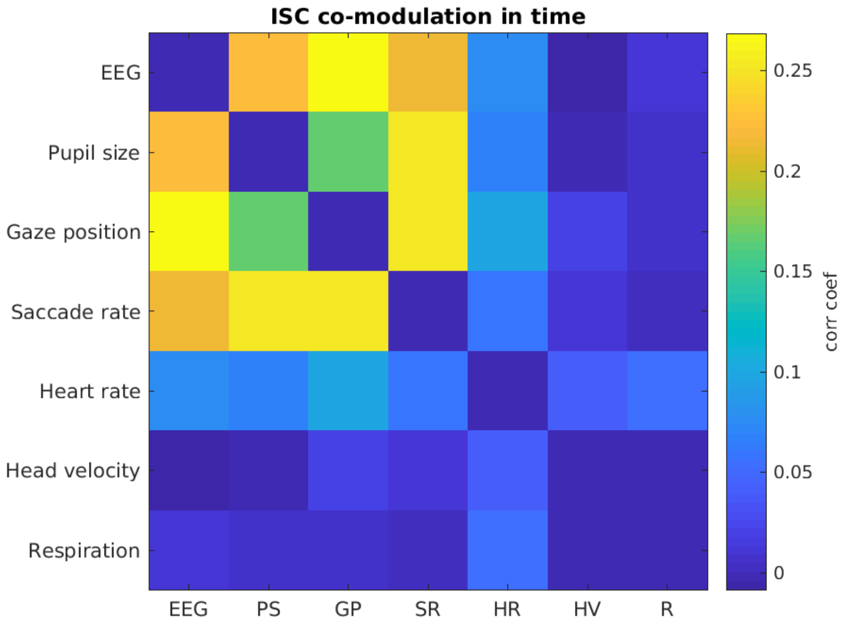

Figure S2: ISC of different modalities correlates weakly across time. ISC is computed here within a sliding time window of 10s width, and correlated between modalities across these time windows (3 video clips lasting 9 min 14 sec) and averaged across subjects (N=43) from Experiment 1. Statistical analysis for a few individual cells is in Fig 3C. Here we are including head velocity and saccade rate, modalities which were tested only after making a prediction based on Fig. 7.

Discussion of Figure 5 for EEG and HR:

For EEG, the coherence spectrum (frequency-resolved ISC, Fig. 5A, first column) is dominant in low frequencies (down to the lowest frequency we measured) and decays substantially once we reach 10Hz. The predominant 10Hz peak in the power spectrum (Fig. 5B, first column) is known as “alpha” activity, and does not appear to correlate across subjects.

For HR, the coherence spectrum (Fig. 5A, second column) has two pronounced peaks that fall within the 0.1 Hz band in the HR power spectrum (Fig. 5B, second column). This band (0.09-0.14Hz) characterized as “low-frequency” in the literature on HR variability [1] is strongly modulated by attention in the present study. Interestingly, this peak is known to be attenuated also during slow-wave sleep.[2] The “high frequency” band (around 0.3Hz) in the power spectrum does not appear to correlate across subjects. This band corresponds to the dominant respiration frequency (see Fig. 3).

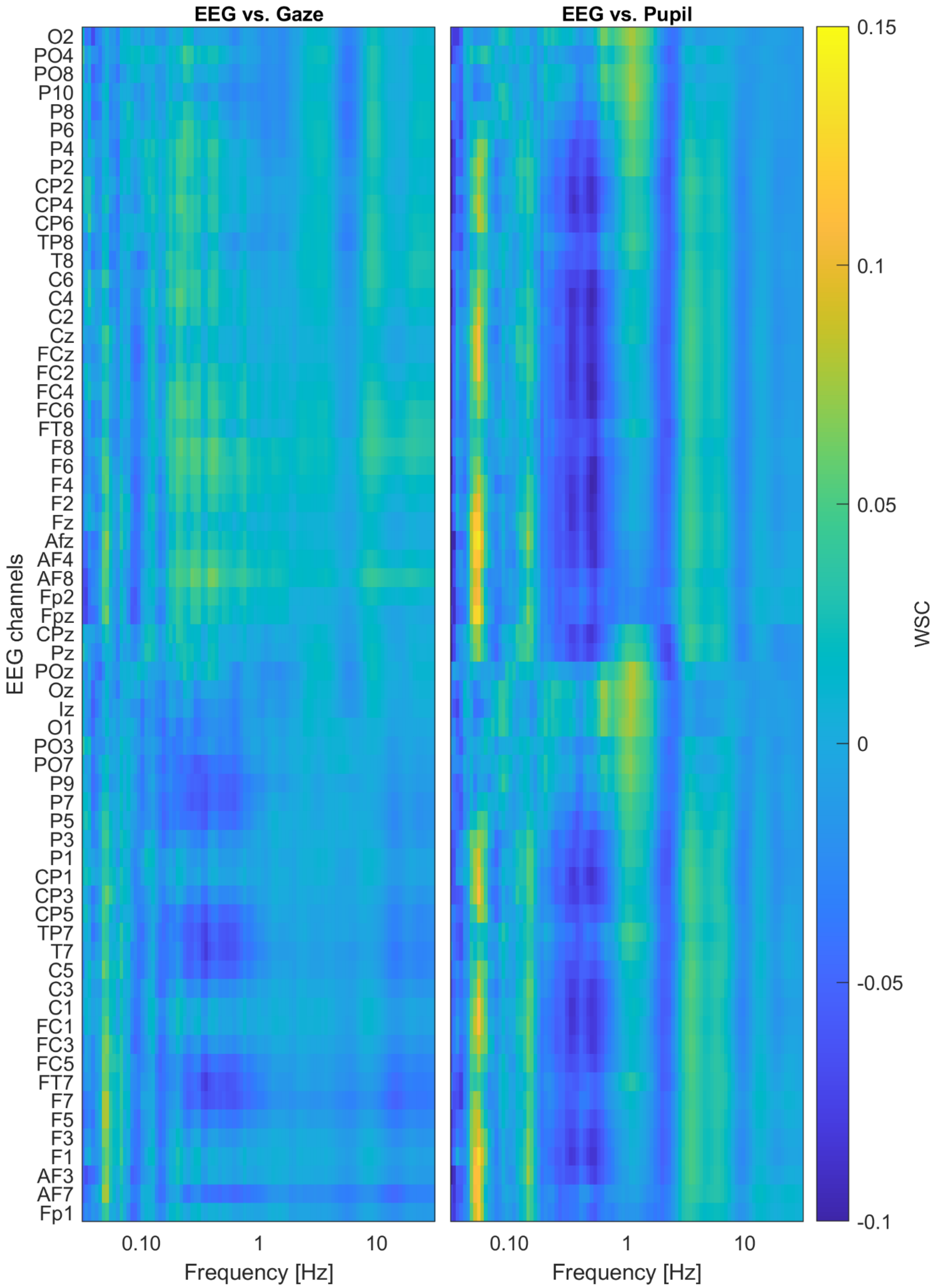

EEG vs. Gaze
EEG vs. Pupil
EEG channels
O2
PO4
PO8
P10
P8
P6
P4
P2
CP2
CP4
CP6
TP8
T8
C6
C4
C2
Cz
FCz
FC2
FC4
FC6
FT8
F8
F6
F4
F2
Fz
Afz
AF4
AF8
Fp2
Fpz
CPz
Pz
POz
Oz
Iz
O1
PO3
PO7
P9
P7
P5
P3
P1
CP1
CP3
CP5
TP7
T7
C5
C3
C1
FC1
FC3
FC5
FT7
F7
F5
F3
F1
AF3
AF7
Fp1
0.10
1
10
Frequency [Hz]
0.15
0.1
0.05
0
-0.05
-0.1
WSC

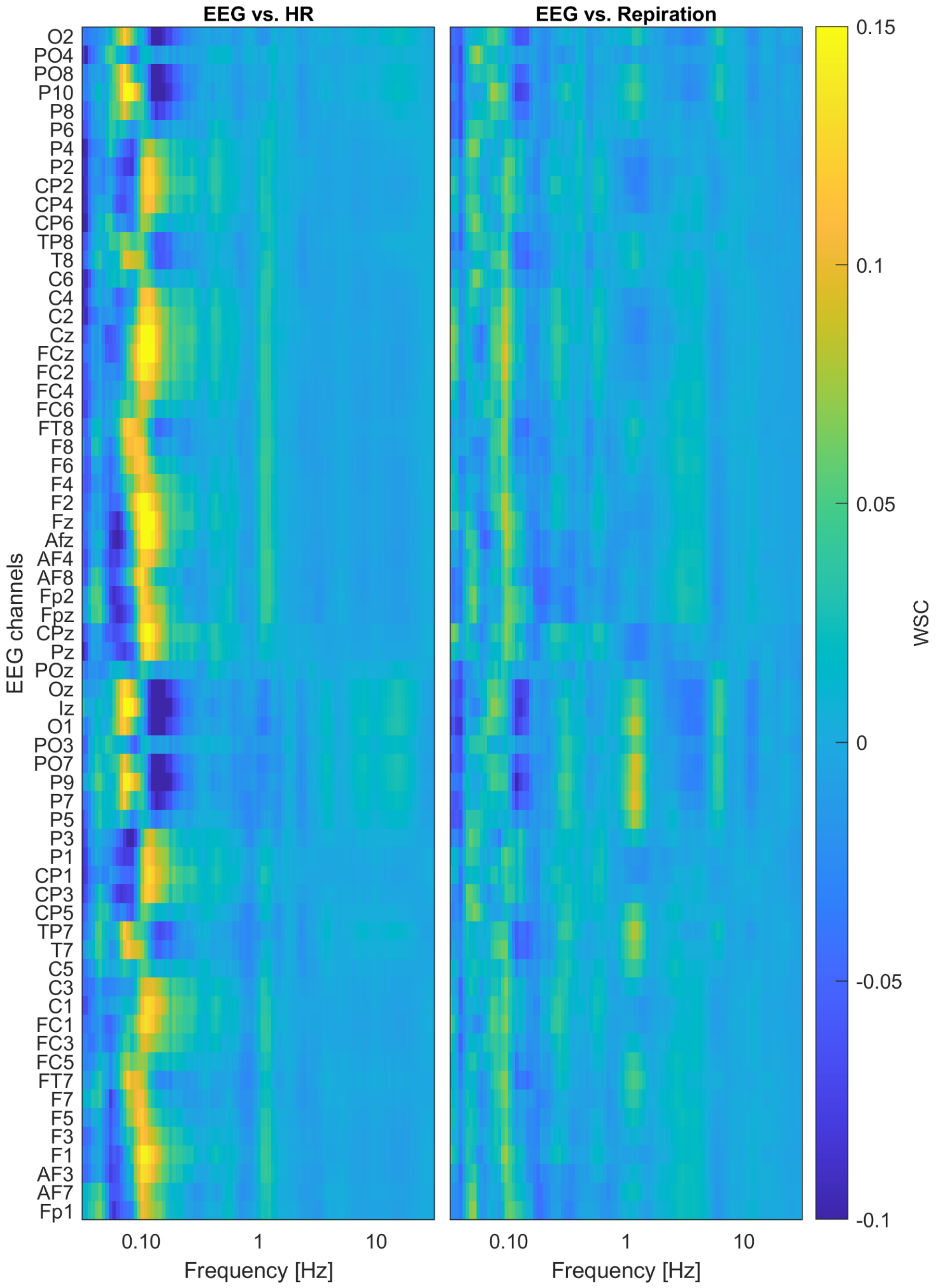

Figure S3: Within-subject correlation between raw EEG and gaze, respiration, pupil and HR signals, resolved by electrode and frequency band. Here correlation is computed as Pearson's

correlation concatenating signals across all videos. It is done separately for each electrode and then band-passed signals.

Conventional indices of arousal and reproducibility of effects

A more specific hypothesis to explain inter-subject correlation or heart rate and pupil size may be that the video causes fluctuations of arousal which are similar across different subjects. To test this we used traditional measures of arousal such as average heart rate (HR) and heart rate variability (HRV), as well as average pupil size, and variation in pupil size. Conventionally, measures of heart rate and pupil are taken on a time scale of minutes, so we computed them for the duration of the videos and averaged cross videos. Overall the results are mixed (Fig. S4): Average pupil size was correlated with ISC of pupil size for Experiment 3 but this did not reproduce for Experiment 2 (Fig. S4A) and we saw a similar pattern with pupil variability. For heart rate and heart rate variability we did not see any significant correlation with ISC in any experiment. Pupil size was modulated by attention (Fig. S4B) but did not predict memory performance in either Experiment 2 or 3 (Fig S4C). Heart rate variability was consistently higher in the distracted condition in both experiments tested (Fig. S4E), which is also consistent with the power spectrum of heart rate (Fig. 5B). Heart rate variability was negatively correlated with memory performance but this was not a robust effect across Experiments 2 and 3 (Fig. S4F). In contrast, the correlation of ISC with memory performance reproduced well for all modalities (Fig. S5). Similarly, the modulation of ISC with attention was replicated for all modalities in Experiment 3 (Fig S6). In view of the robust effects with ISC of pupil size and heart rate, these results indicate that these signals were modulated similarly across subjects on a time scale of 10s or less, whereas for longer time scales of minutes, pupil and heart rate were not meaningfully modulated in the present experimental protocol, except for heart rate variability.

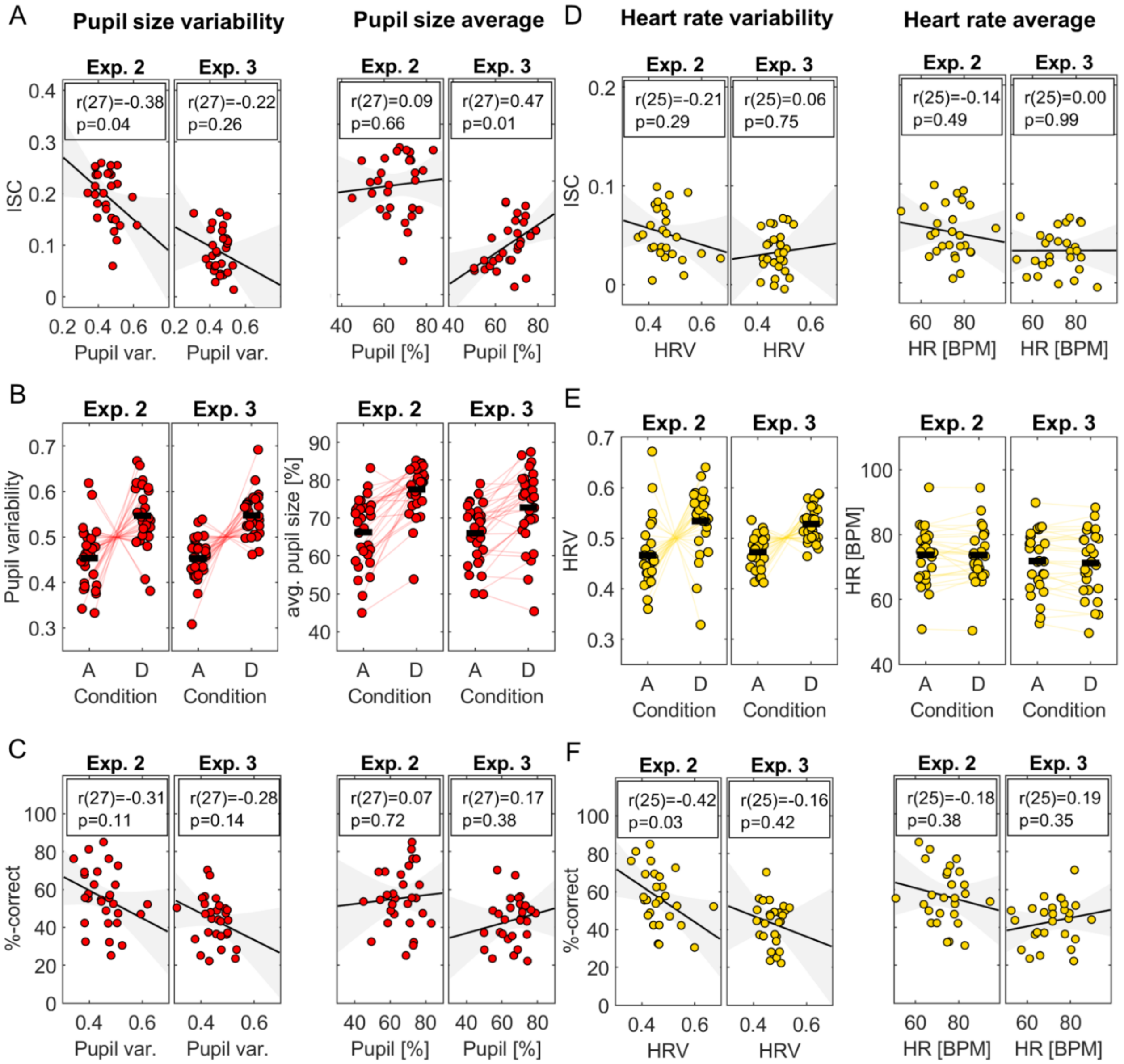

Figure S4: Relation between pupil size and heart rate with memory, ISC and attention: Pupil size, pupil variability, heart rate and heart rate variability were measured for each subject and video clip (time scale of minutes) and averaged over videos for Experiment 2 (Exp. 2) and Experiment 3 (Exp. 3) **A)** average pupil size and pupil variability for each subject compared to the ISC. **B)** Pupil size and pupil variability in the attending and distracted conditions. Lines are connected between the average pupil size of a subject when they watched the 5 videos in an attending condition (A) and distracted conditions (D). **C)** relationship with memory across subjects for pupil size and pupil variability. **D)** same as A) but now for heart rate and heart rate variability **E)** same as B) but now for heart rate and heart rate variability **F)** same as C) but now for heart rate and heart rate variability.

Discussion on conventional measures of pupil and heart rate variability

There is an abundance of studies linking cognitive factors to heart rate variability and pupil responses. These are typically measured on times scales from many seconds to minutes. In our analysis this is captured in the power spectra, which measure the magnitude of fluctuations of HR and pupil size averaged across minutes. In contrast, ISC captures reliable timing of these fluctuations on short time scales (from 30s to less than a second; i.e. the phase of these fluctuations). We are agnostic as to the specific aspect of cognitive processing that reliably drives these fast fluctuations. Indeed, HRV and pupil dilation have been linked to a great number of different cognitive factors. For instance, HRV has been linked to emotions[3], stress[4], attention[5], empathy[6] and other cognitive

factors[7]. Pupil dilation has been linked to arousal[8,9], emotions[10,11], effort[9,12], surprise[13,14], task engagement[15], saliency[16], attention[17] and more. As such, these measures are quite sensitive, but also quite unspecific. We did find an increase in power (i.e. HRV) when subjects are distracted from the videos by a secondary task. Previous studies show that reduced HRV is linked to hyper-vigilance and less flexible attentional control[5]. For the pupil, power captures the magnitude of pupillary response. Previous studies find a pupillary response with shifts in attention[18]. Here, we found an increase in power when viewers are distracted from the stimulus by a secondary mental task. In our view, the traditional measures of heart rate and pupil size variability may be quite sensitive to the specifics of the experimental manipulation. In contrast, the ISC measure gains in robustness by being agnostic to the specific factors elicited by the stimulus. Any of the many factors that might cause a rapid fluctuation in physiological signals will contribute to ISC.

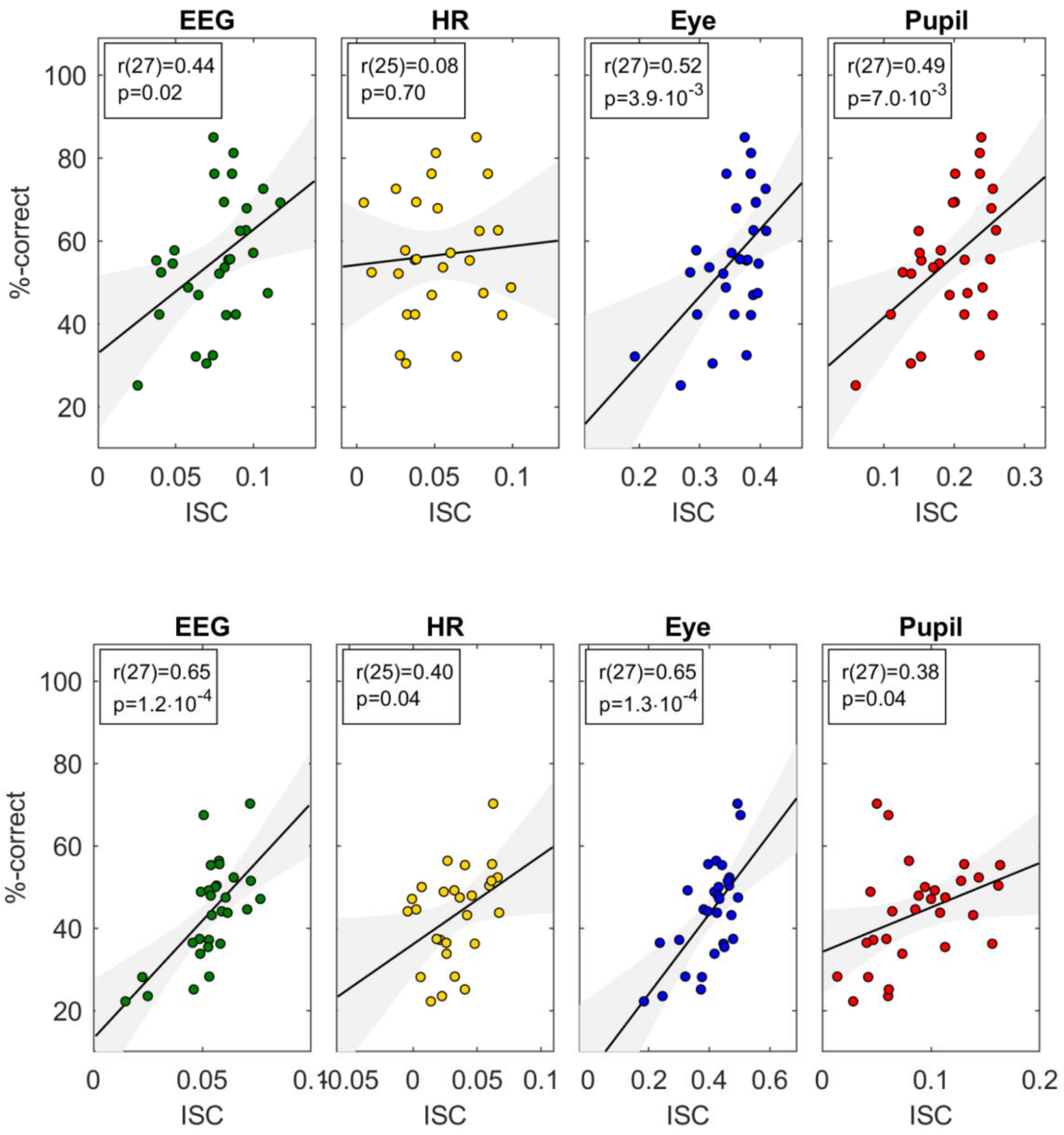

Figure S5: Correlation of ISC with memory and modulation with attention replicates over two additional experiments: First row: Data from Experiment 2 (N=29). For HR 2 subjects were removed due to bad signal quality. Second row: Data Experiment 3 dataset (N=29). For HR 2 subjects were removed due to bad signal quality. Each point is one subject.

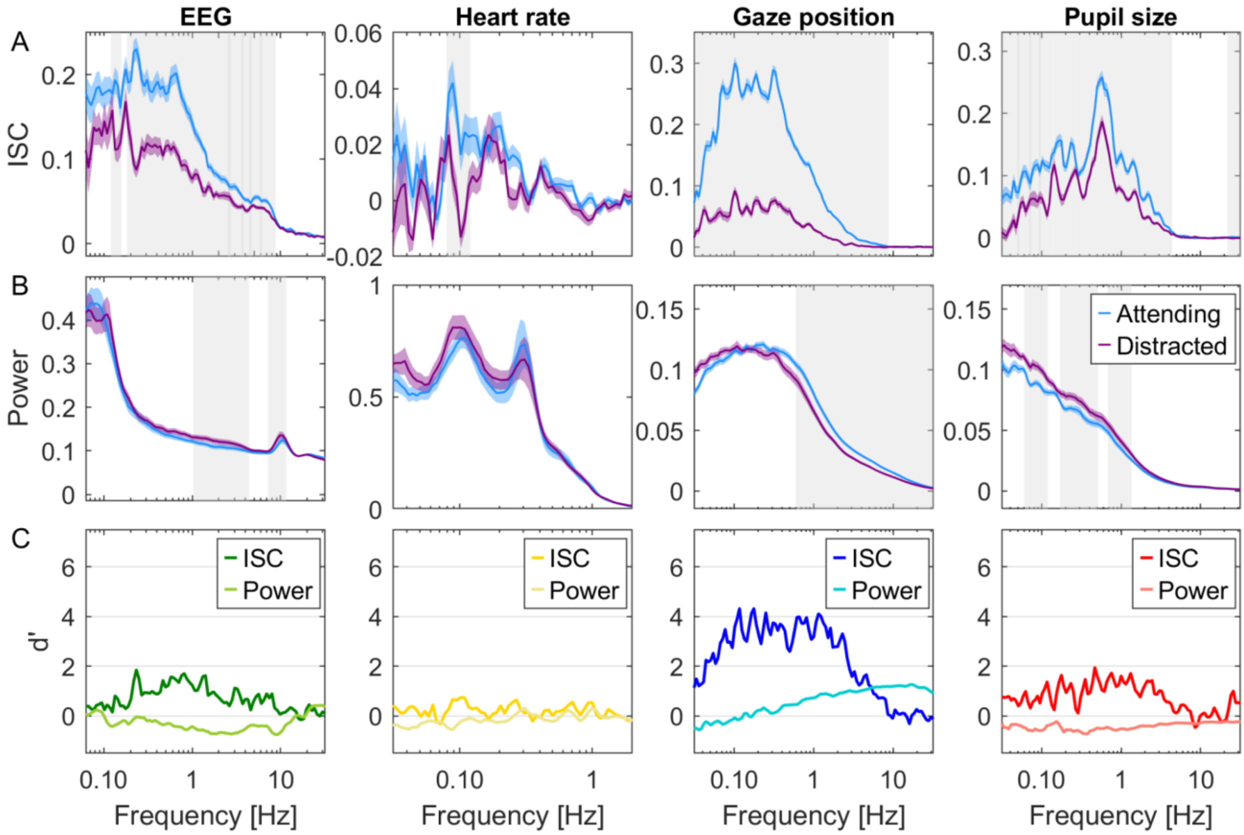

Figure S6. Attentional modulation of ISC and Power resolved by frequency. Same as Fig. 5, but for data from Experiment 3.

Difference in ISC between gender

We have previously shown effects of age and gender for the ISC of EEG. Specifically, a reduction of ISC with age, and a contrast in ISC with sex in the younger viewers, which disappears in adulthood[19]. Thus, we did not expect differences in gender in the present adult population. Consistent with this, we did not find sex differences in ISC for any of the 7 modalities we have tested (Fig. S7) .

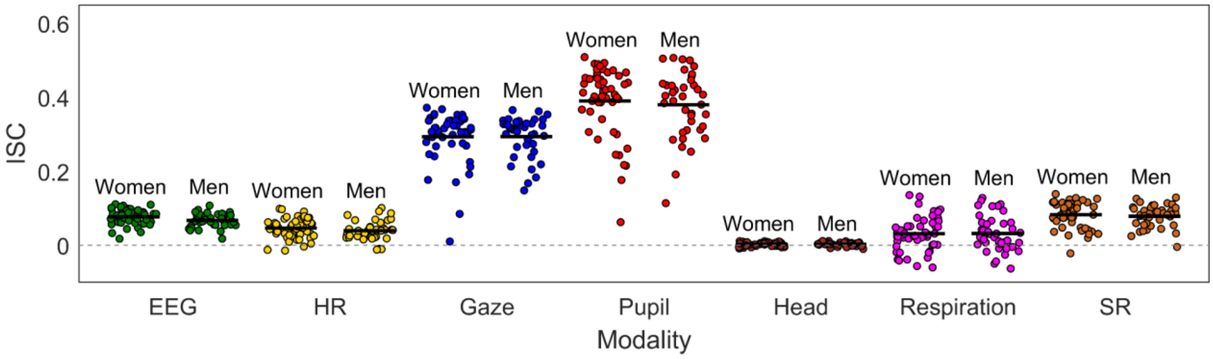

Figure S7. Gender difference in ISC across multiple modalities. ISC is computed jointly for all N=92 participants in Experiment 1, then divided into whether the subject was male or female.

References

1. Malik, M. Heart Rate Variability. *Ann. Noninvasive Electrocardiol.* **1**, 151–181 (1996).

2. Baharav, A. *et al.* Fluctuations in autonomic nervous activity during sleep displayed by power spectrum analysis of heart rate variability. *Neurology* **45**, 1183–1187 (1995).

3. Appelhans, B. M. & Luecken, L. J. Heart Rate Variability as an Index of Regulated Emotional Responding. *Rev. Gen. Psychol.* **10**, 229–240 (2006).

4. Thayer, J. F., Åhs, F., Fredrikson, M., Sollers, J. J. & Wager, T. D. A meta-analysis of heart rate variability and neuroimaging studies: Implications for heart rate variability as a marker of stress and health. *Neurosci. Biobehav. Rev.* **36**, 747–756 (2012).

5. Park, G. & Thayer, J. F. From the heart to the mind: cardiac vagal tone modulates top-down and bottom-up visual perception and attention to emotional stimuli. *Front. Psychol.* **5**, (2014).

6. Kaltwasser, L. *et al.* Sharing the filmic experience - The physiology of socio-emotional processes in the cinema. *PLoS ONE* **14**, (2019).

7. Thayer, J. F. & Lane, R. D. Claude Bernard and the heart–brain connection: Further elaboration of a model of neurovisceral integration. *Neurosci. Biobehav. Rev.* **33**, 81–88 (2009).

8. Hess, E. H. & Polt, J. M. Pupil Size as Related to Interest Value of Visual Stimuli. *Science* **132**, 349–350 (1960).

9. Bradshaw, J. Pupil Size as a Measure of Arousal during Information Processing. *Nature* **216**, 515–516 (1967).

10. Bradley, M. M., Miccoli, L., Escrig, M. A. & Lang, P. J. The pupil as a measure of emotional arousal and autonomic activation. *Psychophysiology* **45**, 602–607 (2008).

11. Partala, T. & Surakka, V. Pupil size variation as an indication of affective processing. *Int. J. Hum.-Comput. Stud.* **59**, 185–198 (2003).

12. van der Wel, P. & van Steenbergen, H. Pupil dilation as an index of effort in cognitive control tasks: A review. *Psychon. Bull. Rev.* **25**, 2005–2015 (2018).

13. Preuschoff, K., 't Hart, B. M. & Einhauser, W. Pupil Dilation Signals Surprise: Evidence for Noradrenaline's Role in Decision Making. *Front. Neurosci.* **5**, (2011).

14. Gee, J. W. de, Correa, C. M. C., Weaver, M., Donner, T. H. & Gaal, S. van. Pupil dilation and the slow wave ERP reflect surprise about choice outcome resulting from intrinsic variability in decision confidence. *bioRxiv* 2020.06.25.164962 (2021) doi:10.1101/2020.06.25.164962.

15. Burlingham, C. S., Mirbagheri, S. & Heeger, D. J. A unified model of the task-evoked pupil response. *bioRxiv* 2021.04.09.439231 (2021) doi:10.1101/2021.04.09.439231.

16. Wang, C.-A. & Munoz, D. P. A circuit for pupil orienting responses: implications for cognitive modulation of pupil size. *Curr. Opin. Neurobiol.* **33**, 134–140 (2015).

17. Konishi, M., Brown, K., Battaglini, L. & Smallwood, J. When attention wanders: Pupillometric signatures of fluctuations in external attention. *Cognition* **168**, 16–26 (2017).

18. Mathôt, S. & Van der Stigchel, S. New Light on the Mind's Eye: The Pupillary Light Response as Active Vision. *Curr. Dir. Psychol. Sci.* **24**, 374–378 (2015).

19. Petroni, A. *et al.* The Variability of Neural Responses to Naturalistic Videos Change with Age and Sex. *eNeuro* **5**, ENEURO.0244-17.2017 (2018).
